# Biodiversity, Distribution, and Conservation of Plants and Fungi: Effects of Global Warming and Environmental Stress

**DOI:** 10.3390/jof8050441

**Published:** 2022-04-24

**Authors:** Alona Yu. Biketova, Rodica Catana, Anush Kosakyan

**Affiliations:** 1 Jodrell Laboratory, Royal Botanic Gardens, Kew, Richmond TW9 3DS, UK; 2 Mycological Society of Israel, P.O. Box 164, Pardesiya 42815, Israel; 3 Institute of Biology Bucharest of Romanian Academy, 296 Splaiul Independentei, 060031 Bucharest, Romania; rodica.catana@ibiol.ro; 4 Institute of Parasitology, Biology Centre, Czech Academy of Sciences, 37005 Ceske Budejovice, Czech Republic; anna.kosakyan@gmail.com

The estimation of global biodiversity and its conservation is an old, but still unresolved, concern in biology. On the one hand, the number of described species is constantly increasing, especially with the accumulation of modern morphological and molecular data; on the other hand, the existence of many species is threatened due to environmental and anthropogenic pressures.

Most of the articles in this Special Issue are devoted to the diversity, taxonomy and molecular phylogeny of fungi [1,2,3,4,5,6]. Ten new species and three new taxonomic combinations are described for science [1,2,3,5,6]. It is noteworthy that half of these manuscripts are devoted to three genera of the family *Boletaceae*—one of the most remarkable and most vulnerable groups of fungi to the destruction of ecosystems. Novel comprehensive phylogenetic and taxonomic analyses of *Leccinum*, *Hemileccinum*, *Exsudoporus*, *Amoenoboletus*, and allied genera, together with descriptions of eight new species and two taxonomic combinations, and the typification of *Exsudoporus floridanus* indicate that there are many unresolved issues, even related to such a relatively well-studied group of macroscopic fungi as *Boletaceae* [2,3,6]. Mešić et al. (2021) described a new agaricoid species *Inocybe brijunica*, growing in the Mediterranean Biogeographical Region, one of the most prominent global climate change hot spots [1]. Two other articles presented investigations of the biodiversity of the *Ascomycota* genera *Calonectria* and *Wickerhamomyces* [4,5].

Another group of manuscripts was dedicated to the ecological, physiological, and applied aspects of mycobiota [7,8,9,10,11]. Jabborova et al. (2021) studied the interactions between biochar and arbuscular mycorrhizal fungi (AMF) and spinach. It was shown that these fungi can promote plant growth, improve soil properties, and maintain microbial activity [7]. A review by Boorboori and Zhang (2022) provided comprehensive up-to-date information on the use of AMF in the phytoremediation of arsenic, cadmium, lead, and chromium [8]. Another study was devoted to the composition of major, trace, and rare-earth elements in 15 different species of wild edible mushrooms. The data obtained did not indicate a significant exposure to anthropogenic influences, regardless of the sampling location. While the contents of major elements seem to be influenced by species–specific affinities, this is not true for trace elements, whose contents probably reflect the geochemical characteristics of the sampling site [9].

Adamo et al. (2021) conducted a metabarcoding analysis of ectomycorrhizal fungi in five different Mediterranean pine forests (*Pinus nigra*, *P. halepensis*, *P. sylvestris,* and two mixed) and concluded that fungal communities did not differ in phylogenetic composition, structure, or phylogenetic diversity among tree hosts [10]. Mihai et al. (2022) offered bio-friendly solutions to reduce the waste of coconut coir, pine sawdust, and paper (as some of the main pollutants in Ecuador) by using them as suitable growth substrates for the edible fungus *Pleurotus ostreatus*. The results showed that all waste products represent desirable substrates for fungal growth, with an emphasis on coconut coir waste, whose usage increased the desirable characteristics of the fungi [11].

The editors of the Special Issue express their gratitude to all the authors who contributed to this issue as well as to MDPI’s staff for their valuable support and prompt decisions.
